# Predicting the incidence of brucellosis in Western Iran using Markov switching model

**DOI:** 10.1186/s13104-020-05415-5

**Published:** 2021-03-01

**Authors:** Maryam Mohammadian-Khoshnoud, Majid Sadeghifar, Zahra Cheraghi, Zahra Hosseinkhani

**Affiliations:** 1 Department of Biostatistics, School of Public Health, Hamadan University of Medical Sciences, Hamadan, Iran; 2 Department of Statistics, Faculty of Science, Bu-Ali Sina University, Hamadan, Iran; 3 Department of Epidemiology, School of Public Health, Hamadan University of Medical Sciences, Hamadan, Iran; 4 Modeling of Noncommunicable Diseases Research Center, Hamadan University of Medical Sciences, Hamadan, Iran; 5 Metabolic Diseases Research Center, Research Institute for Prevention of Non-Communicable Diseases, Qazvin University of Medical Sciences, Qazvin, Iran

## Abstract

**Objective:**

Brucellosis is a zoonosis almost chronic disease. Brucellosis bacteria can remain in the environment for a long time. Thus, climate irregularities could pave the way for the survival of the bacterium brucellosis. Brucellosis is more common in men 25 to 29 years of age, in the western provinces, and in the spring months. The aim of this study is to investigate the effect of climatic factors as well as predicting the incidence of brucellosis in Qazvin province using the Markov switching model (MSM). This study is a secondary study of data collected from 2010 to 2019 in Qazvin province. The data include brucellosis cases and climatic parameters. Two state MSM with time lags of 0, 1 and 2 was fitted to the data. The Bayesian information criterion (BIC) was used to evaluate the models.

**Results:**

According to the BIC, the two-state MSM with a 1-month lag is a suitable model. The month, the average-wind-speed, the minimum-temperature have a positive effect on the number of brucellosis, the age and rainfall have a negative effect. The results show that the probability of an outbreak for the third month of 2019 is 0.30%.

## Introduction

Brucellosis is a zoonosis almost chronic disease which is transmitted by direct or indirect contact with infected animals or products [1]. *B. melitensis* is the most common and acute pathogen that can cause various symptoms such as sudden tremors, general body aches [2–4]. Despite a good health care system in Iran, brucellosis is still an important endemic disease. Iran ranks fourth in the world of the incidence of brucellosis [5–7]. According to the National Guidelines, epidemiological and clinical data of patients in Iran are recorded online in the health monitoring system. Patients with epidemiological and clinical signs of brucellosis, i.e. fever, myalgia, results of two laboratory tests are considered as cases. Laboratory tests include the Wright test (> 1.8 indicating infection) and the 2ME (Mercaptoethanol Brucella agglutination test).

In Iran, the incidence of brucellosis is reported to be between 50 and 500 per 100,000 people, often of the *B. melitensis* type and is known as a local disease. The incidence rate of brucellosis in Qazvin is 27.43 per 100,000 during 2010–2019 [8].

In epidemiology, a sudden increase in the incidence of a disease at a specific time or place is called an outbreak. In this study, the outbreak of the disease means a sudden increase in the disease in Qazvin. Climatic factors directly and indirectly affect the growth and reproduction dynamics of pets, sheep/goat human interactions, pathogen replication and population immunity affect brucellosis ecology [9–12]. Temperature changes may have a greater impact on the brucellosis epidemic than other climatic factors [13].

Despite the significant effect of climatic factors on brucellosis, these factors have been overlooked in most studies. The primary purpose of this study was to investigate the effect of climatic factors on the incidence of brucellosis. The second purpose of this study is to use MSM for prediction. A number of researchers have used the MSM in disease prediction [14] but so far the performance of this method has not been evaluated in brucellosis data.

## Main text

### Method

This study is a secondary study of data collected from April 2010 to March 2019 in Qazvin, which is extracted from the database of the Qazvin University of Medical Sciences and the meteorological system of the province [8]. No individual data were used. The available information was cumulative without mentioning personal information.

The response variable is the number of brucellosis cases. Month, rural-ratio, age, men-ratio, the ratio of contact with livestock, Non-pasteurized dairy and factors in the meteorological system include average-monthly-temperature, total-monthly-rainfall, average-wind-speed, maximum-monthly-temperature, minimum-monthly-temperature and wind-speed are considered as explanatory variables. The number of cases of brucellosis is very low in some months and if we consider daily or weekly cases, the number of cases will be zero on some times. Also, because brucellosis bacteria survive in the environment for a long time, considering monthly cases will help to better understand the factors affecting brucellosis.

In this study, the number of monthly cases of human brucellosis in Qazvin is a dependent variable (Y_t_). Independent variables (X_t_s) with time lags of 0, − 1, and − 2 were considered in two-states and three-states models. ${{\varvec{\sigma}}}_{{\varvec{s}}{\varvec{t}}}$ is the standard deviation of the error in the model.

MSM including time series {Y_t_}_t=1,…,T_ and a sequence of related variables x_1_,…,x_T_ is introduced with the relation between x_t_ and Y_t_ as follows:

$${Y}_{t}={{\varvec{f}}}^{({{\varvec{s}}}_{{\varvec{t}}})}\left({{\varvec{x}}}_{{\varvec{t}}}\right)+{{\varvec{\sigma}}}_{{\varvec{s}}{\varvec{t}}}{{\varvec{\epsilon}}}_{{\varvec{t}}}$$

where ${{\varvec{\epsilon}}}_{{\varvec{t}}}$ has a standard normal distribution and S_t_ is the state at time t of a non-observable N state Markov chain.

According to the fitted MSM, beta is the estimation of the effect of each variable on the response in each state. SE is the standard deviation of beta and p-value shows the significance of each variable in the relevant state.

In two-state models, if we define state 1 as the disease outbreak period and 2 state as the non-outbreak period, the probability of an outbreak in period t + 1 can be as follows [14]:

$$P\left({s}_{t+1}=1\right)=\frac{(1-{p}_{22})}{(2-{p}_{11}-{p}_{22})}$$

$$P\left({s}_{t+1}=2\right)=\frac{(1-{p}_{11})}{(2-{p}_{11}-{p}_{22})}$$

A two-state MSM with switching all effects is considered. The data were analyzed through SPSS software version 26 and MSwM package of R software 3.6.3 [15].

### Results

From 2010 to 2019, 3194 people were infected with brucellosis. Of these, the highest incidence related to the year 2015 with 512 (16%), and the lowest incidence related to the year 2010 with 192 (6%). In matching the seasons with the Gregorian months, spring is related to March, April and May, summer is related to June, July and August, fall is related to September, October, and November and finally, winter is related to December, January and February. Among seasons, the summer with 961 (30.1%) and winter with 805 (25.2%) have the highest number of infected, respectively. The highest number of patients is related to temperatures 26 ℃ (6.6%), 25 ℃ (6.4%). The highest number of infected people is related to zero rainfall of 444 cases (13.9%) and the lowest number of patients is related to the average-monthly-rainfall of 17.8 (0.2%) and 64.5 (0.2%). The lowest number of patients is related to the average-wind-speed of 0.7 with 6 cases (0.2%).

#### Fitting MSM

**Fig. 1:**
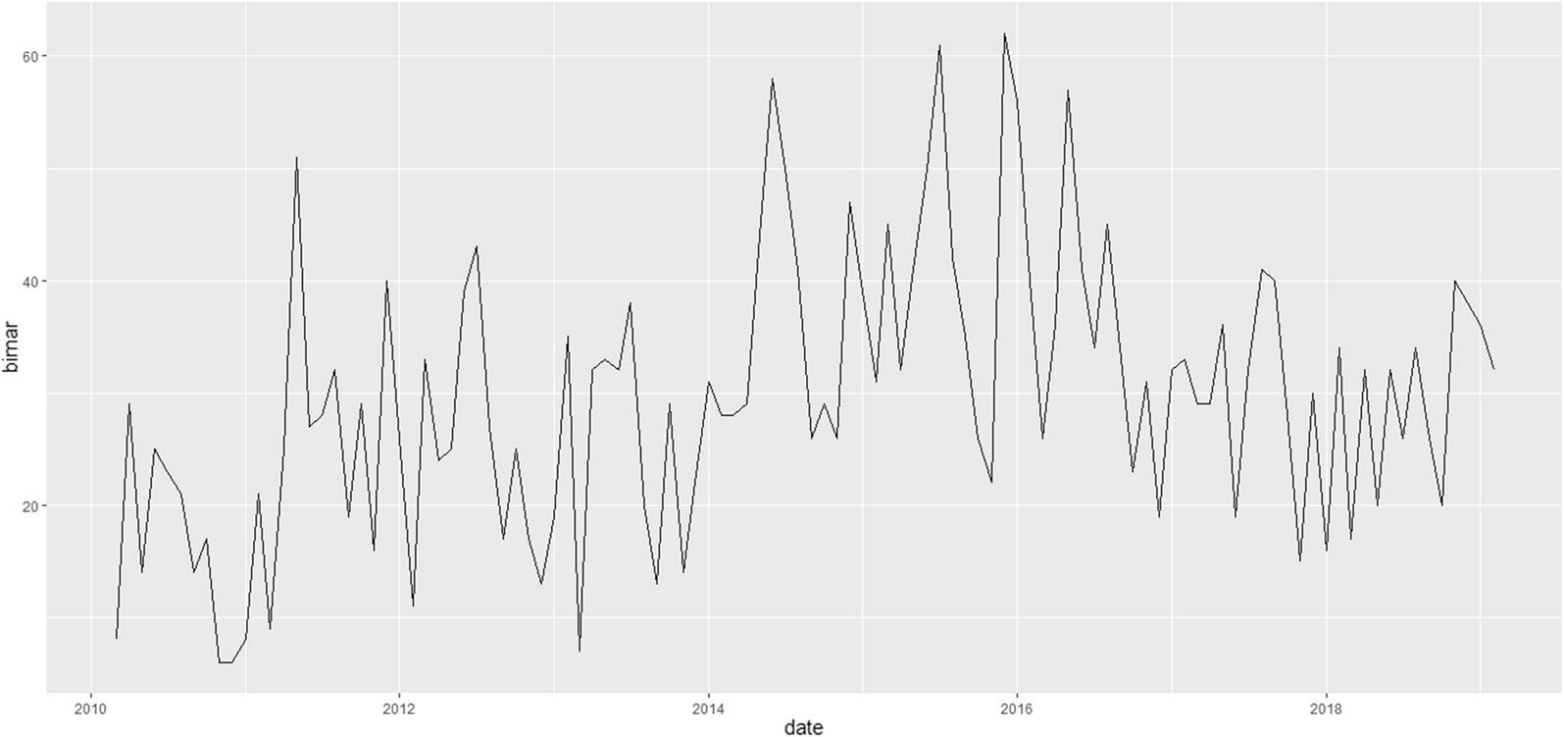
The monthly trend chart for the number of brucellosis cases from 2010 to 2018

The MSM was fitted with two and three states and both models were fitted with 0, − 1, and − 2 lags for climate variables. Temporal lag is defined as the time interval between climatic characteristics and the incidence of brucellosis. Based on a comparison between models, based on BIC, two-state MSM with a time lag of − 1 is suitable. For this reason, only this model is offered to provide more results (Fig. 1).

Age, month, rural-ratio, men-ratio, Non-pasteurized dairy, average-monthly-temperature, total-monthly-rainfall, average-wind-speed, maximum-monthly-temperature, Minimum-monthly-temperature, average-wind-speed, ratio of contact with livestock were recognized as significant variables (Table 1).

**Table 1:** The fit of the two-state markov switching model with time lags of 0, − 1, − 2

Variables (state)	MSM with two states								
	Lag 0			Lag − 1			Lag − 2		
	B	SE	p-value	B	SE	p-value	B	SE	p-value
Intercept (1)	3.274	3.073	0.287	9.981	11.531	0.387	33.426	11.879	0.005
Intercept (2)	10.01	11.628	0.389	3.254	3.047	0.286	− 2.162	14.711	0.883
Age (1)	− 0.152	0.016	0	− 0.116	0.118	0.326	− 0.25	0.059	0
Age (2)	− 0.116	0.118	0.327	− 0.152	0.016	0	0.499	0.224	0.026
Month (1)	1.618	0.108	0	0.817	0.371	0.028	1.164	0.408	0.004
Month (2)	0.816	0.371	0.028	1.618	0.108	0	− 0.045	0.585	0.939
Rural ratio (1)	3.172	0.113	0	− 0.287	0.389	0.461	2.666	0.513	0
Rural ratio (2)	− 0.287	0.389	0.461	3.173	0.113	0	0.164	0.365	0.653
Men ratio (1)	2.92	0.079	0	− 3.009	0.665	0	− 3.013	0.608	0
Men ratio (2)	− 3.012	0.667	0	2.928	0.1	0	1.412	1.517	0.352
Non-pasteurized dairy (1)	− 0.57	0.07	0	0.569	0.229	0.013	1.056	0.296	0
Non-pasteurized dairy (2)	0.57	0.229	0.0127	− 0.571	0.069	0	− 0.273	0.23	0.235
Average monthly temperature (1)	− 4.223	0.135	0	0.241	0.82	0.769	0.935	0.859	0.276
Average monthly temperature (2)	0.239	0.852	0.779	− 4.229	0.142	0	− 2.546	1.017	0.012
Total monthly rainfall (1)	− 0.038	0.015	0.012	− 0.034	0.041	0.405	− 0.242	0.048	0
Total monthly rainfall (2)	− 0.034	0.041	0.405	− 0.038	0.015	0.011	0.009	0.053	0.85
Average wind speed (1)	1.509	0.214	0	19.024	2.848	0	18.097	3.285	0
Average wind speed (2)	19.017	2.869	0	1.533	0.185	0	5.163	3.336	0.122
Maximum monthly temperature (1)	1.717	0.119	0	− 0.177	0.567	0.754	− 1.377	0.597	0.021
Maximum monthly temperature (2)	− 0.176	0.585	0.763	1.718	0.118	0	0.722	0.629	0.251
Minimum monthly temperature (1)	3.203	0.096	0	0.231	0.408	0.572	0.345	0.403	0.393
Minimum monthly temperature (2)	0.231	0.417	0.579	3.207	0.101	0	2.289	0.599	0
Wind speed (1)	0.944	0.072	0	− 0.698	0.301	0.02	− 0.433	0.25	0.083
Wind speed (2)	− 0.699	0.302	0.02	0.944	0.072	0	0.469	0.288	0.103
Ratio of contact with livestock (1)	− 0.387	0.059	0	0.904	0.28	0.001	0.283	0.221	0.2
Ratio of contact with livestock (2)	0.904	0.28	0.001	− 0.387	0.059	0	1.212	0.561	0.031

Autocorrelation and partial autocorrelation of residual and squared residual for model is confirmed lack of autocorrelation in the residual and the model seems to fit logically and there's no serial dependency on the residual.

**Fig. 2:**
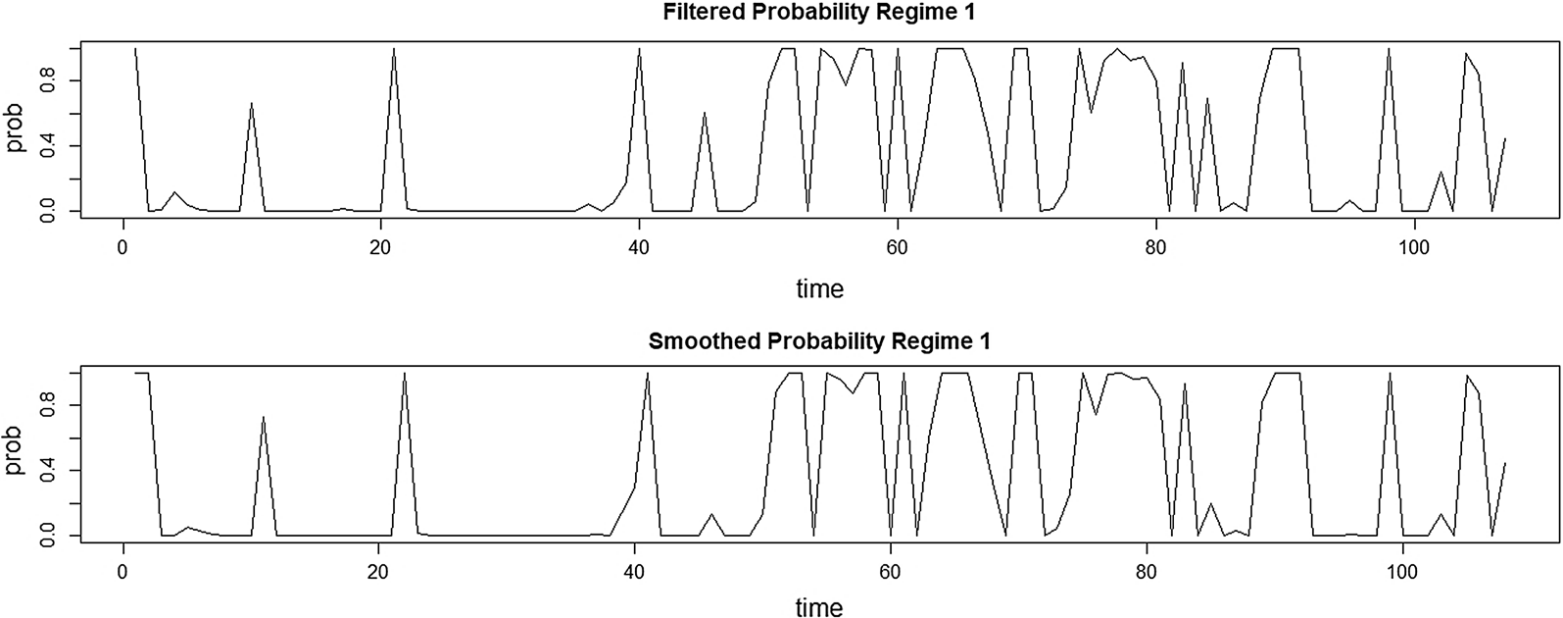
Probability of smoothed and filtered states for state of one and two

Figure 2 shows the smoothed and filtered probabilities for state of one and two. Smoothed probabilities are used to determine peaks and depressions and 0.5 is determined as the cut-off value for 1 and 2 states. The filtered probabilities are calculated using the first observation up to t and the smoothed probabilities are calculated using the total observations.

The Q–Q plot shows where the normality hypothesis is questionable for series. Transition probability matrix in MSM as follows:

$$\left[\begin{array}{cc}{P}_{11}& {P}_{12}\\ {P}_{21}& {P}_{22}\end{array}\right]=\left[\begin{array}{cc}{P}_{11}& {1-P}_{11}\\ {1-P}_{22}& {P}_{22}\end{array}\right]=\left[\begin{array}{cc}0.72& 0.28\\ 0.65& 0.35\end{array}\right]$$

where the probability of non-outbreak state in both t and t + 1 periods is 0.72, the probability of changing series from non-outbreak state in period t to outbreak state in period t + 1 is 0.28 and the probability of the series changing from the outbreak state in period t to non-outbreak state in period t + 1 is 0.65. When we are in a non-outbreak state (0.72), the process tends to stay the same state and the process is transferred to the outbreak state with a probability of 0.28.

The probability of an outbreak in t + 1 is as follows:

$$P\left[{S}_{t+1}=1\right]=\frac{1-0.35}{2-0.72-0.35}=0.70$$

$$P\left[{S}_{t+1}=2\right]=0.30$$

Since the data is up to the second month of 2019, the probability of an outbreak for the third month of 2019 (1 month later) is very low and is equal to 0.30%.

The biggest difference between the coefficients of the variables in two states is related to the average-wind-speed. Therefore, the average-wind-speed is the most important factor in incidence brucellosis.

Month, average-wind-speed and minimum-temperature coefficients are positive which indicate a positive effect on the number of brucellosis. The age and total-monthly-rainfall coefficients are negative, indicating a negative effect on the number of brucellosis.

The temporal changes of the observed cases of brucellosis and the values estimated by the MSM are illustrated in Additional file 1: Figure S1. The model has a relatively good performance in identifying peaks incidence of brucellosis.

### Discussion

The highest incidence of brucellosis is related to 2015 with 512 cases (16%). The average wind speed was 1.89. The incidence is the highest at zero total-monthly-rainfall. Total-monthly-rainfall was 0 at 444 days (13.9%), which includes most days of study. The minimum-temperature was − 1 and the maximum-temperature was 30 ℃. The mean age of the patients was about 38 years.

There is no clear pattern in the number of cases of brucellosis in the 8 years studied and fluctuations in the incidence of this disease can be seen with three peaks 2015 December, July 2015, June 2014. These results are inconsistent with the results of Lee's study. In the Lee’s study, the incidence of human brucellosis in South Korea peaked in September 2006 and has dropped dramatically which indicates effective eradication [16]. In Rafiemanesh’s study, the incidence of brucellosis decreased from 2007 to 2016 which indicates an increase in the coverage of prevention programs, especially livestock vaccination [17]. These results are inconsistent with the results of the present study.

In the 2010 year, the lowest number of cases has been reported, followed by an upward trend until 2011. The reason for the rising trend of the disease from 2010 to 2011 may be related to the improvement of the data registration in the country's health system. This result is consistent with Hashtarkhani’s study [18]. From 2014 to 2017, there is an upward trend in the number of brucellosis cases which is inconsistent with Hashtarkhani's study. In the study of Hashtarkhani after 1990, we see a decreasing trend in the incidence of the disease [18]. The results of the study show that there are the highest number of infected people in summer and winter seasons. These results are consistent with the results of the Tapak’s study. The results of the Tapak’s study show that hot summers and cold winters make the disease less common while climate moderation in these seasons exacerbates the disease [19]. Therefore, the temperate climate of these seasons in Qazvin increases the number of patients with brucellosis. Model fit results indicate the negative effect of age and total-monthly-rainfall on the number of brucellosis. This result is consistent with the results of Entezari's study. The results of the Entezari's study indicate a negative relationship between rainfall and brucellosis. In fact, as the rainfall decreases, the number of infected people increases [20].

In zenoses, changes in climatic factors naturally affect the contamination rate and dynamics of animal hosts as well as human exposure to infected animals [21].

High levels of evaporation and sun exposure cause drought and limit the germination of the plant, while dry environments may cause human skin dryness and cracked skin and increase the risk [13].

Comparing the coefficients of explanatory variables in two state, the average-wind-speed is the most important factor in incidence brucellosis. This result is consistent with the results of Ahmadkhani’s study. The results of Ahmadkhani's study indicate a positive correlation between wind-speed, temperature, greenness and incidence of brucellosis [22]. The results of the present study are inconsistent with those of Tapak. According to the Tapak’s study, the wind at high speeds reduces the disease. This is because the bacterium has a shorter lifespan in the air.

The mean age of the patients was about 38 years. This result has been confirmed in other studies [23, 24]. The results of this study indicate that P_11_ and P_22_ are larger than P_21_ and P_12_, respectively. In other words, states do not tend to change. That is, when we are in a non-outbreak state, the process tends to remain the same state. Also, P_21_ is higher than P_12_, which indicates that prolonged periods of non-outbreak lead to a reduction in the probability of outbreak during the year.

As the country's health progresses in many areas, the incidence of brucellosis is expected to decline. However, the results of the present study indicate a sharp increase in the disease between 2014 and 2017, which requires a lot of health attention in Qazvin province. The necessary learning for high-risk age and occupational groups and not consuming unpasteurized dairy products and not having contact with suspicious animals and cooperating with livestock vaccination should be on the agenda of the region's health institutions.

## Conclusion

The MSM can be used to detect factors related to the incidence of brucellosis as well as to predict the incidence of brucellosis. Most climatic parameters were effective in incidence the disease, and the most influential factor was the average-wind-speed. The probability of disease outbreak in the third month of 2019 was predicted to be 0.30%.

## Limitation

One of the limitations of this study is the limited period of the time series data and lack of daily information. Another limitation is the lack of comparison between different time series models.

## Supplementary Information

**Additional file 1.** Figure S1: Prediction values obtained using Markov switching model along with the observed values.

## Data Availability

The dataset used and/or analysed during the current study are available from the Zahra Hosseinkhani on reasonable request.

## Abbreviation

MSM: Markov switching model
