# Supplementary material for: Predicting the incidence of brucellosis in Western Iran using Markov switching model
Source: BMC Res Notes. 2021 Mar 1;14:79. doi: 10.1186/s13104-020-05415-5 (PMC7923320; doi:10.1186/s13104-020-05415-5)
Supplement: Supplementary file 1 — Additional file 1. Figure S1: Prediction values obtained using Markov switching model along with the observed values. [file 13104_2020_5415_MOESM1_ESM.docx]

**Introduction:**

The disease has disappeared in many developed countries such as Australia, Japan, but in the Mediterranean, Middle East, and parts of Asia is still as a major public health problem(5).

**Methods:**

Markov switching model was first introduced by Quandt (1972) and Quandt and Goldfeld (1973) and then developed by Hamilton (1989) to extract commercial cycles(24). Nonlinear models are divided into two groups in terms of the velocity of change from one state to another. In models such as STAR and artificial network, the velocity of change from one state to another is slow and gentle. In some other models, this transfer is done quickly, and the Markov switching model is one of these models(24). This model is also called the switching regime model(25). Markov switching model belongs to the family of state-space models. In these models, there are two types of measurement and transfer equations. The measurement equation defines how the hidden states affect the unobserved random variables and the transfer equation defines how state variables evolve over time. The observed random variables in Markov switching depend on historical values as well as latent state variables. This position of the Markov switching model is suitable for more time-series issues(25).

A feature of the Markov switching model is that the switching mechanism is controlled by the unobserved state variable. This latent state variable follows a first-order Markov chain. The Markov feature regulate that the current value of the status variable depends on the previous value. This model is suitable for describing correlated data that shows dynamic patterns over different time periods.

**Method of estimating the parameters of Markov switching**

Estimation of the Markov switching model is divided into two main stages(27). In the first step, we assume that the states are known. In practice, S is not known, but this division simplifies the discussion, and the results obtained are later used in repetitive estimation methods(27). Estimation of the defining parameters of the model and implementation of the non-observed state process is obtained by the maximum likelihood estimate method. Due to the large number of parameters required for estimation, standard nonlinear optimization methods can be unstable(28). An EM algorithm is used to prevent this and also because the status of S_t_ is hidden(28). Finally, we estimate the S_t_ and model parameters.

**Choosing the number of status**

Choosing the suitable number of states is not easy. Even for the simplest of Markov's parametric switching models, there are different criteria for selecting the number of states. These criteria include the AKaike information criterion, the Bayesian information criterion, the Hannan-Quinn criterion, and cross-validated likelihood (26). In practice, meaningful interpretation of states plays an important role in the selection of the number k(27).

**Statistical inference of state variable**

An important application of Markov switching models is to make inferences about the state of S_t_ provided the data and model(27). x is the observations available at time t and model M(27). There are two types of statistical inference about S_t_ in practice(27). The first type which is related to the probability of P(S_t_=i I x^t^,M), is called the probability of filtered status, where i is to determine the status of the Markov switching model(27). The second type of inference is related to the probability of P(S_t_=i I x^T^, M) and is called the probability of a smoothed state where T is the sample size and T> t(27). The criterion for placing each observation in each of the regimes is determined based on the probability related to that observation and comparing it with a 50% probability. Smoothed probabilities are used to determine peaks and depressions and 0.5 is determined as the cut-off value for zero and one states. The filtered probabilities are calculated using the first observation up to t and the smoothed probabilities are calculated using the total observations.

Figure S1- Prediction values obtained using Markov switching model along with the observed values
